# Appendicitis caused by the metastasis of HER2-positive breast cancer

**DOI:** 10.1186/s40792-016-0235-5

**Published:** 2016-09-28

**Authors:** Ryutaro Mori, Manabu Futamura, Kasumi Morimitsu, Kazuhiro Yoshida

**Affiliations:** Department of Surgical Oncology, Graduate School of Medicine, Gifu University, 1-1 Yanagido, Gifu, 501-1194 Japan

**Keywords:** Secondary appendiceal neoplasm, Appendicitis, Lobular carcinoma

## Abstract

The appropriate therapy for metastatic breast cancer must be selected based on the immunohistochemical phenotype of the cancer. However, biopsy for metastatic lesions is difficult. We herein report a patient with incidental appendicitis caused by a metastatic breast cancer which was successfully treated with effective therapy chosen based on the pathological diagnosis obtained on resection. The patient was a 56-year-old female with right breast cancer and an immunohistochemical status of estrogen receptor (ER) (+), progesterone receptor (PgR) (+), human epidermal growth factor receptor 2 (HER2) (3+), and Ki67 40 %. She received epirubicin and cyclophosphamide therapy followed by docetaxel and trastuzumab, and total mastectomy with axillary dissection was performed. Thereafter, she received postmastectomy radiation, adjuvant trastuzumab, and adjuvant hormone therapy with letrozole. One year and 8 months after the operation, she developed right hydronephrosis and swollen para-aortic lymph nodes and her hormone therapy was changed to fulvestrant therapy. However, she additionally developed left hydronephrosis and multiple bone metastases, and pertuzumab, trastuzumab, and docetaxel therapy was started. After six cycles, her disease became well-controlled, and maintenance with pertuzumab and trastuzumab was introduced. However, after another 7 months, she developed new vertebral metastasis and acute appendicitis and laparoscopic appendectomy was performed. A pathological investigation of the resected appendix revealed some clusters of atypical cells in the subserosa and muscle layer, which showed an immunohistochemical status of ER (+), PgR (−), HER2 (3+), and E-cadherin (−). These findings led to the diagnosis as appendiceal metastasis of invasive lobular carcinoma (ILC) from the breast. Thereafter, she received trastuzumab-DM1 and her disease was well-controlled again. Appendicitis caused by breast cancer is very rare. However, ILC sometimes develops metastases in the abdominal cavity; an appendiceal tumor should therefore be included in the differential diagnosis. A pathological diagnosis of metastatic tumor could be very useful for selecting the effective therapy.

## Background

The appropriate therapy for metastatic breast cancer must be selected based on the immunohistochemical phenotype of the cancer, such as the estrogen receptor (ER) status and human epidermal growth factor 2 (HER2) status. Hormone therapy is indicated for ER-positive cases, and anti-HER2 therapy is restricted to HER2-positive cases. However, in many cases, therapy for metastatic breast cancer is determined based on the immunohistochemical phenotype of the primary site resected in a radical operation, as biopsy for metastatic lesions is difficult. If the selected therapy has no effect, another therapy must be tried, or a pathological diagnosis for the metastatic lesion must be obtained via a difficult technique, such as computed tomography (CT)-guided biopsy, bone biopsy, or laparotomic biopsy, since the immunohistochemical phenotype can occasionally differ between the primary and metastatic lesions [1].

We herein report a patient with incidental appendicitis caused by a metastatic breast cancer which was successfully treated with effective therapy chosen based on the pathological diagnosis obtained on resection.

## Case presentation

**Fig. 1:**
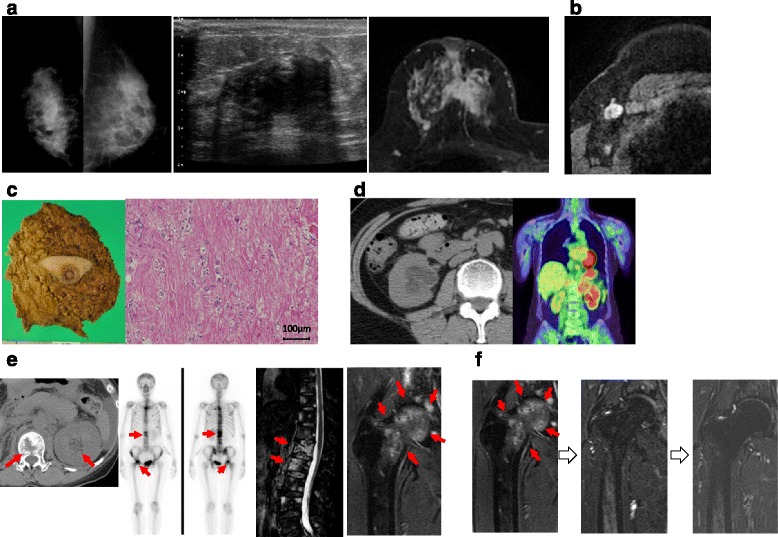
The images and surgical specimens obtained before the patient developed acute appendicitis. **a** Breast tumor on mammography, US, and MRI at the first visit. **b** Axillary lymph nodes on MRI at the first visit. **c** The surgical specimen obtained at mastectomy. **d** Right hydronephrosis and swollen para-aortic lymph nodes on CT and PET. **e** Left hydronephrosis and bone metastases on CT, PET, and MRI. **f** Right femoral bone metastasis after the start of pertuzumab, trastuzumab, and docetaxel therapy on MRI

The patient was a 56-year-old female with no remarkable medical history. She visited our hospital with a chief complaint of a right breast mass in April 2011. The mass in the upper medial portion of her right breast was a 3-cm-diameter tumor with an unclear border on mammography, ultrasonography (US), and magnetic resonance imaging (MRI) (Fig. 1a), and the lymph nodes in the axilla and subclavian were swollen (Fig. 1b). The results of a histological examination for a core needle biopsy specimen were a breast cancer (invasive ductal carcinoma) with an immunohistochemical status of ER (+), progesterone receptor (PgR) (+), HER2 (3+), and Ki67 40 %. She received four cycles of epirubicin and cyclophosphamide therapy followed by four cycles of docetaxel and trastuzumab as preoperative chemotherapy, and total mastectomy with axillary dissection was performed in December 2011 (Fig. 1c). A postoperative pathological investigation revealed that almost all of the tumor cells on the resected breast and lymph nodes had disappeared. Thereafter, she received postmastectomy radiation therapy for the chest wall and supraclavicular region, adjuvant trastuzumab for 1 year, and adjuvant hormone therapy with letrozole.

One year and 8 months after the operation, she developed right hydronephrosis and swollen para-aortic lymph nodes (Fig. 1d) and her hormone therapy was changed to fulvestrant therapy. However, she additionally developed left hydronephrosis and multiple bone metastases in the skull, right acetabulum, and right iliac crest 5 months after the therapy was changed (Fig. 1e). Pertuzumab, trastuzumab, and docetaxel therapy was started after left nephrostomy, and after six cycles of the therapy, the bone metastases had shrunk (Fig. 1f) and her bilateral hydronephrosis was relieved. Her disease seemed to be well-controlled. Therefore, maintenance therapy with pertuzumab and trastuzumab (without docetaxel) was introduced and the nephrostomy tube was removed.

**Fig. 2:**
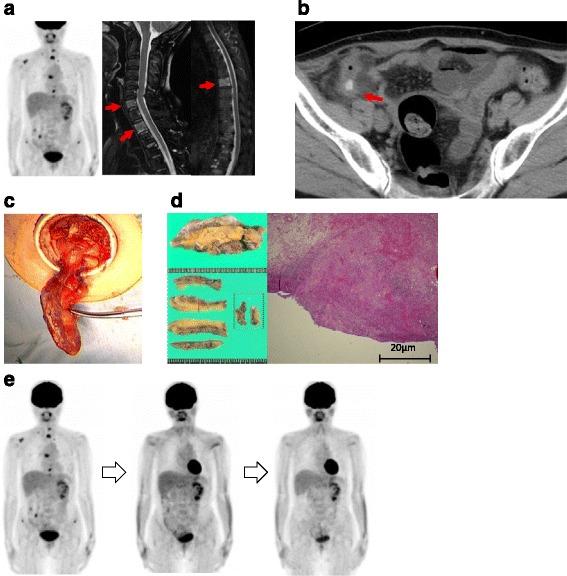
The images at the time of development and after the patient developed acute appendicitis. **a** New vertebral metastasis on PET and MRI. **b** Swollen appendix on axial and coronal CT. **c** An image taken during laparoscopic appendectomy. **d** A specimen obtained at appendectomy. **e** PET images after the start of trastuzumab-DM1

Seven months after the start of maintenance therapy, she developed new vertebral metastasis (Fig. 2a) and visited the emergency ward at our hospital complaining of right lower abdominal pain. Computed tomography showed a swollen appendix with fecal stones (Fig. 2b), and a blood test revealed elevated white blood cells (16,270/μl) and CRP (7.07 mg/dl), suggesting acute appendicitis (Fig. 2b). Laparoscopic appendectomy was performed (Fig. 2c). The pathological findings showed infiltration of neutrophils within the submucosa and subserosa, which were consistent with gangrenous appendicitis. Further investigation of the specimen revealed some clusters of atypical cells in the subserosa and muscle layer (Fig. 2d).

**Fig. 3:**
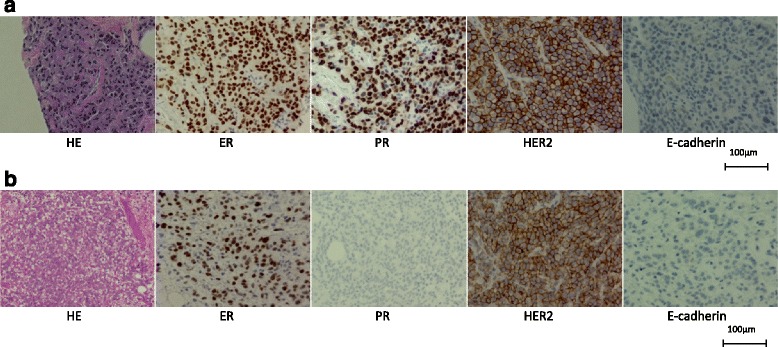
The histopathological and immunohistochemical diagnoses of the breast tumor and appendiceal tumor. **a** The breast tumor findings (HE, ER, PR, HER2, E-cadherin). **b** The appendiceal tumor findings (HE, ER, PR, HER2, E-cadherin)

An immunohistochemical analysis of the breast tumor showed ER (+), PgR (+), HER2 (3+), and E-cadherin (−) (Fig. 3a), while that of the appendiceal tumor showed ER (+), PgR (−), HER2 (3+), and E-cadherin (−) (Fig. 3b). Although the expression of PgR differed between the breast tumor and appendiceal tumor, both tumors had similar morphologic features, overexpression of HER2, and loss of E-cadherin, and we therefore concluded that the appendiceal tumor was a metastasis from invasive lobular carcinoma (ILC) of the breast.

Thereafter, she received trastuzumab-DM1 therapy, since the appendiceal tumor still had overexpression of HER2, after which the metastatic tumors detected on PET/CT almost disappeared again (Fig. 2e).

### Discussion

We experienced having a patient with appendicitis caused by a metastasis of ILC. Thanks to the immunohistochemical analysis of the resected appendiceal tumor, we succeeded in continuing effective anti-HER2 therapy.

ILC of the breast accounts for 14 % of all breast cancer cases [2] and presents with a loss of E-cadherin expression [3]. It can metastasize to the abdominal cavity as peritoneal dissemination, para-aortic lymph node swelling, and small bowel metastasis. Arpino et al. reported that ILC and invasive ductal carcinoma (IDC) develop such metastases in 6.7 and 1.8 % of cases, respectively [4]. In our case, we failed to obtain the histological diagnosis of ILC at the initial therapy session. However, the metastatic pattern of her disease, such as para-aortic lymph node swelling and hydronephrosis, made us suspect ILC, and we succeeded in detecting carcinoma cells that were E-cadherin (−) in the resected appendix.

Connor et al. reported that incidental appendiceal tumor detected after appendectomy accounts for only 0.9 % of cases (74/7990), and among them, metastatic tumor was only found in 11 cases [5]. Yoon et al. previously reported 139 cases with metastatic appendiceal tumors, with primary sites mainly consisting of ovary (56 cases), colon (35 cases), and stomach (7 cases). No cases of appendiceal metastasis from breast cancer were described in their study [6], indicating that appendicitis caused by the metastasis of breast cancer is very rare.

HER2 expression can sometimes differ between the primary and metastatic tumors. Amir et al. reported that an HER2-positive primary tumor and HER2-negative metastatic tumor account for 12.5 % of cases, while an HER2-negative primary tumor and HER2-positive metastatic tumor account for 4.6 % of cases [1]. We must therefore perform biopsy of the metastatic tumor if anti-HER2 therapy for metastatic breast cancer is less effective than expected. In the present case, her metastatic tumor seemed to show resistance to pertuzumab and trastuzumab therapy, and we could not have selected an appropriate subsequent therapy without an immunohistochemical analysis of the resected appendix.

Basic research investigating the efficacy of trastuzumab-DM1 for HER2-positive breast cancer cell lines has found that trastuzumab-resistant cells retain sensitivity to trastuzumab-DM1 [7]. Furthermore, the efficacy of trastuzumab-DM1 was found to be related to the strength of HER2 expression, and trastuzumab-DM1 was not effective against HER2-negative cells [8]. These results suggest that trastuzumab-DM1 is worth trying even in cases with trastuzumab-resistant tumors, and biopsy for metastatic tumors is useful for confirming the strength of HER2 expression.

## Conclusions

Appendicitis caused by breast cancer is very rare. However, ILC sometimes develops metastases in the abdominal cavity, and we therefore should suspect an appendiceal tumor, as in the present patient. A pathological diagnosis of metastatic tumor could be very useful for selecting the most effective subsequent therapy.

## Abbreviations

CT: Computed tomography
ER: Estrogen receptor
HER2: Human epidermal growth factor 2 (HER2)
ILC: Invasive lobular carcinoma
MRI: Magnetic resonance imaging
PgR: Progesterone receptor
US: Ultrasonography

## References

[1] Amir E, Clemons M, Purdie CA, Miller N, Quinlan P, Geddie W, Coleman RE, Freedman OC, Jordan LB, Thompson AM (2012). Tissue confirmation of disease recurrence in breast cancer patients: pooled analysis of multi-centre, multi-disciplinary prospective studies. Cancer Treat Rev.

[2] Martinez V, Azzopardi JG (1979). Invasive lobular carcinoma of the breast: incidence and variants. Histopathology.

[3] Lehr HA, Folpe A, Yaziji H, Kommoss F, Gown AM (2000). Cytokeratin 8 immunostaining pattern and E-cadherin expression distinguish lobular from ductal breast carcinoma. Am J Clin Pathol.

[4] Arpino G, Bardou VJ, Clark GM, Elledge RM (2004). Infiltrating lobular carcinoma of the breast: tumor characteristics and clinical outcome. Breast Cancer Res.

[5] Connor SJ, Hanna GB, Frizelle FA (1998). Appendiceal tumors: retrospective clinicopathologic analysis of appendiceal tumors from 7,970 appendectomies. Dis Colon Rectum.

[6] Yoon WJ, Yoon YB, Kim YJ, Ryu JK, Kim YT (2010). Secondary appendiceal tumors: a review of 139 cases. Gut Liver.

[7] Barok M, Tanner M, Köninki K, Isola J (2011). Trastuzumab-DM1 causes tumour growth inhibition by mitotic catastrophe in trastuzumab-resistant breast cancer cells in vivo. Breast Cancer Res.

[8] van der Lee MM, Groothuis PG, Ubink R, van der Vleuten MA, van Achterberg TA, Loosveld EM, Damming D, Jacobs DC, Rouwette M, Egging DF, van den Dobbelsteen D, Beusker PH, Goedings P, Verheijden GF, Lemmens JM, Timmers M, Dokter WH (2015). The preclinical profile of the duocarmycin-based HER2-targeting ADC SYD985 predicts for clinical benefit in low HER2-expressing breast cancers. Mol Cancer Ther.

